# Linking glucocorticoid-induced osteoporosis to osteoimmunology

**DOI:** 10.1038/s41419-020-03250-x

**Published:** 2020-12-14

**Authors:** Stephan von Gunten, Hans-Uwe Simon

**Affiliations:** Institute of Pharmacology, University of Bern, Bern, Switzerland

**Keywords:** Cell biology, Immunology

Over millions of years, the skeletal and the immune systems have coevolved in the development from bony fish to terrestrial animals to form a symbiotic and highly interactive relationship. It has been suggested that environmental pressures, such as higher levels of UV light and oxygen, or lower levels of calcium in the terrestrial environment, promoted the establishment of the protective endosteal niche for haematopoietic stem cells (HSCs) in the bone marrow^1^. Besides other aspects, the tight connection between the immune and the skeletal systems is reflected by the following facts: (1) origination of osteoclasts from hematopoietic progenitor cells, (2) colocalization of osteoblast and osteoclast progenitor cells with immune cell progenitor and memory cells in the bone marrow, (3) immunomodulatory effects of the major pro-osteoclastogenic cytokine receptor activator of NF-κB ligand (RANKL) and its expression by both osteoblast lineage cells and lymphocytes, (4) reciprocal effects of immune and bone remodelling cells in cell differentiation and bone remodelling, and (5) reduction of bone mass in inflammatory disorders, eventually as a consequence of excessive bone resorption^2^. In the last two decades, significant new insights into the complex interaction between the immune and skeletal systems brought light to the research field, which was referred to as ‘osteoimmunology’ by Arron and Choi in 2000^3^.

Glucocorticoids (GCs) exhibit a plethora of genomic and non-genomic effects in different tissues^4^, and significantly influence both bone remodelling and immune cells. High GC exposure, as it occurs in steroid therapy, has catabolic effects on bones and can result in osteoporosis. In fact, GC-induced osteoporosis (GIOP) is the most frequent cause of secondary osteoporosis^5^. The pharmacological effects of exogenous GCs in osteoporosis induction remain only partly understood, yet may include negative effects on differentiation, proliferation, survival, and function of osteoblasts and osteocytes, eventually involving Wnt signalling pathways, the transcription factors AP-1 and Notch, as well as specific miRNAs^5^. Besides direct effects on osteoclasts and their precursors, GCs may stimulate osteoclastogenesis by induction of RANKL and reduce expression of osteoprotegerin (OPG), a decoy receptor of RANKL, in osteoblastic cells and osteocytes^6–8^.

**Fig. 1:**
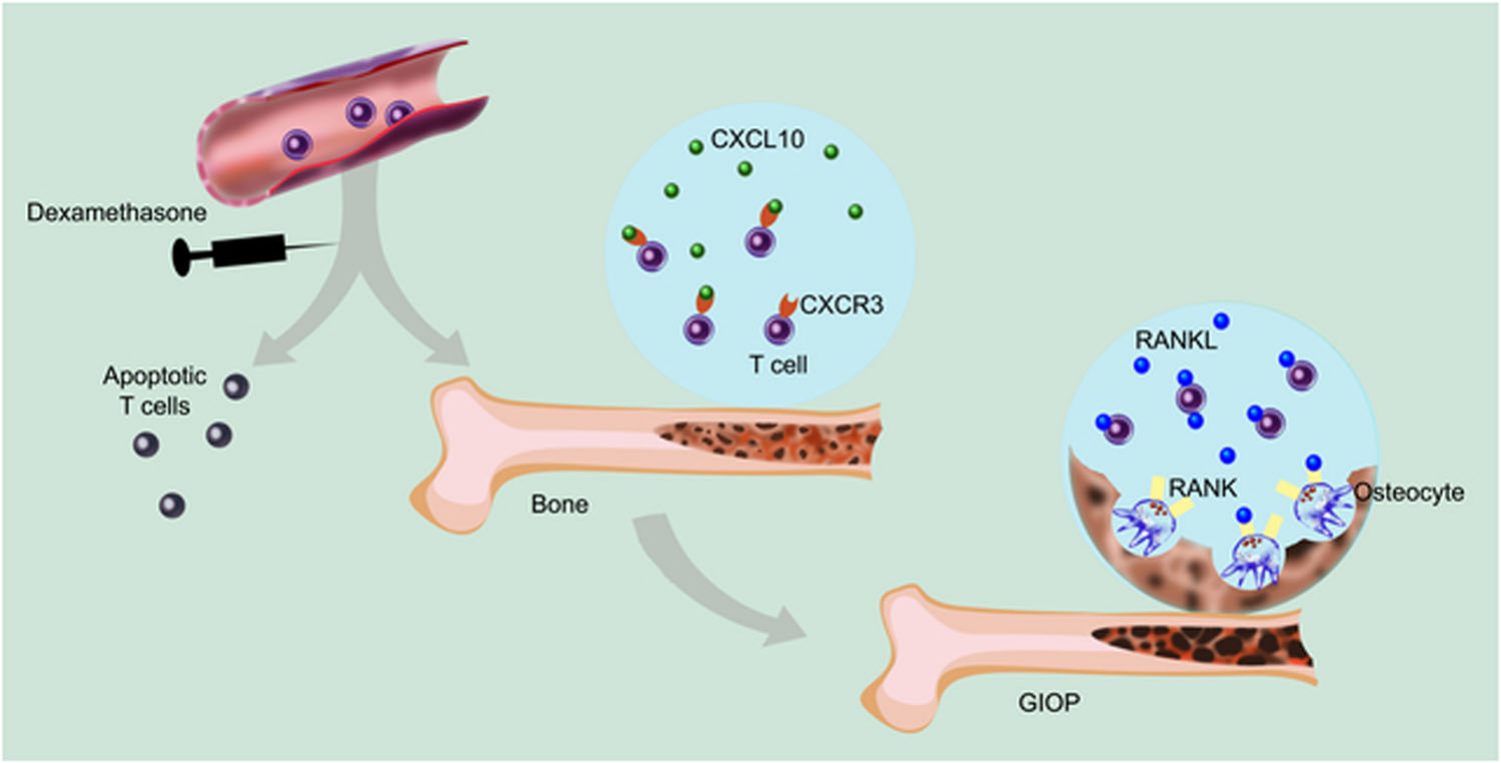
Proposed involvement of T cells in glucocorticoid-induced osteoporosis. While peripheral T cells may undergo apoptosis upon exposure to dexamethasone, some T cells accumulate in the bone marrow in a CXCL10-CXCR3 axis-dependent manner. Latter T cells are protected from cell death and promote RANKL-induced osteoclastogenesis. Illustration by Aldona von Gunten. GIOP glucocorticoid-induced osteoporosis, RANK receptor activator of NF-κB, RANKL RANK ligand.

In this issue of *Cell Death and Disease*, Song et al.^9^ report a central role of T cells for GIOP (Fig. 1). Using models with T-cell-deficient SCID or nude mice, they demonstrated that T cells are indispensable for the establishment of GIOP. SCID mice develop osteoporosis upon adoptive transfer of T cells, which was paralleled by an increase of RANKL in serum. T cells homing in the bone marrow were found to express RANKL and were able to stimulate ex vivo the differentiation of osteoclasts in co-culture experiments with myeloid RAW264.7 cells. Given that in other types of osteoporosis T-cell-derived cytokines have been shown to enhance RANKL expression in osteoblasts and other cells^10,11^, it is possible that such indirect effects also contribute mechanistically to the development of GIOP.

Peripheral lymphopenia can result from impaired lymphopoiesis in the endosteal niche due to diminished IL-7 production by osteoblasts, as observed under septic conditions^12^. In contrast, while dexamethasone treatment resulted in the reduction of circulating T-cell numbers and an increase of apoptotic T cells in the spleen, Song et al.^9^ observed an accumulation of viable T cells in the bone marrow, suggesting a protective influence of the endosteal niche. The increased T-cell homing to the bone marrow was found to be dependent on chemokine ligand receptor interactions with significant involvement of the CXCL10-CXCR3 axis. CXCL10 and CXCR3 receptor signalling have previously been linked to bone loss related to increased osteoclast differentiation and activity in various models of disease^2^, including conditions with an established pathogenic role of T cells.

The study by Song et al. highlights the importance of T cells in the pathogenesis of GIOP and may support the consideration of osteoimmunological approaches in the prevention of GIOP. However, while the existing literature documents distinct contributions of T-cell subsets, cytokines, and chemokines in the development of osteoporosis^1,10,11^, their relevance to GIOP remain to be explored. Furthermore, it will be important to consider that significant differences in immune responses exist not only between species^13,14^, but also among human individuals^15,16^. Future pharmacotherapeutic strategies are expected to be inspired by a better understanding of molecular networks^17^, and the mutual interactions between the bone and immune systems in GIOP, eventually resulting in more personalized approaches to steroid therapy.
